# Brain response pattern identification of fMRI data using a particle swarm optimization-based approach

**DOI:** 10.1007/s40708-016-0049-z

**Published:** 2016-04-07

**Authors:** Xinpei Ma, Chun-An Chou, Hiroki Sayama, Wanpracha Art Chaovalitwongse

**Affiliations:** 1 Department of Systems Science & Industrial Engineering, Binghamton University, the State University of New York, Binghamton, USA; 2 Departments of Industrial & Systems Engineering, Department of Radiology, Integrated Brain Imaging Center, University of Washington, Seattle, USA; 3 Department of Radiology, Integrated Brain Imaging Center, University of Washington, Seattle, USA

**Keywords:** Brain response pattern, Brain functional connectivity, Pattern classification, Particle swarm optimization, Feature selection, Interaction selection

## Abstract

Many neuroscience studies have been devoted to understand brain neural responses correlating to cognition using functional magnetic resonance imaging (fMRI). In contrast to univariate analysis to identify response patterns, it is shown that multi-voxel pattern analysis (MVPA) of fMRI data becomes a relatively effective approach using machine learning techniques in the recent literature. MVPA can be considered as a multi-objective pattern classification problem with the aim to optimize response patterns, in which informative voxels interacting with each other are selected, achieving high classification accuracy associated with cognitive stimulus conditions. To solve the problem, we propose a feature interaction detection framework, integrating hierarchical heterogeneous particle swarm optimization and support vector machines, for voxel selection in MVPA. In the proposed approach, we first select the most informative voxels and then identify a response pattern based on the connectivity of the selected voxels. The effectiveness of the proposed approach was examined for the Haxby’s dataset of object-level representations. The computational results demonstrated higher classification accuracy by the extracted response patterns, compared to state-of-the-art feature selection algorithms, such as forward selection and backward selection.

## Introduction

Functional magnetic resonance imaging (fMRI) is one of the publicly used neuroimaging techniques to capture brain neural activity in small volumetric units (called voxels) in the brain by measuring the change of blood-oxygen-level dependent (BOLD) signals over time. Broadly speaking, it has advanced the understanding of brain functional activity by fMRI in various cognitive and behavioral neuroscience applications, such as Alzheimer’s disease [1], aging [2], autism [3], depression [4], schizophrenia [5], and attention-deficit hyperactivity disorder [6]. The overarching goal of these research studies with fMRI is to examine and understand the brain states among different regions of interest (ROI) associated with specific brain functions or disorders, so that treatments and interventions can be made precisely according to stimulus or diagnostic conditions [7].

Conventionally, univariate analysis of fMRI data was widely used to identify the ROIs of brain functions (i.e., localization) by statistical tests on individual voxels in most research studies [8, 9]. In more recent years, multi-voxel pattern analysis (MVPA) of fMRI data has been increasingly applied to identify response patterns of voxels as a whole [3, 10, 11]. The MVPA can be modeled as a high-dimensional pattern classification problem to train a classification (or prediction) model based on the fMRI BOLD signals, in which voxels (as features) are identified in response to stimulus or diagnostic conditions (as class labels). In most neuroscience experimental studies, the number of stimulus samples is relatively much less than the number of voxels in the brain. This leads to a computational challenge of high feature-to-sample ratio from the machine learning viewpoint [12]. Therefore, various advanced feature selection and sparse optimization techniques were proposed to enhance the computational results in terms of classification efficacy and informativeness of selected voxels [13–17]. It leads to two-fold objectives: (1) it aims to select a minimum number of voxels included in classification models and (2) the classification accuracy needs to be maximized.

Technically, a number of computational approaches have been proposed and employed to solve this multi-objective high-dimensional problem [18–21]. Computational intelligence-based approaches, such as genetic algorithms (GA), simulated annealing (SA), ant colony optimization (ACO), and particle swarm optimization (PSO), are at the forefront of this research [22–26]. They are implemented in conjunction with a classifier to find a set of highly representative features for classification tasks. Heuristic feature selection approaches stand out in terms of theoretical simplicity, strong global search ability, and less expensive computational cost. Instead of exhaustively exploring the solution space, these algorithms adopt effective learning schemes to optimize the feature selection [27]. In addition, heuristic approaches pay more attention to find the best combination of features rather than evaluating the goodness of features individually. These benefits of computational intelligence approaches indicate a great potential in analyzing brain response patterns of high-dimensional fMRI data [28].

However, when solving high-dimensional optimization problems where multiple local optima exist, most classical heuristic optimization algorithms fail to find (near) global optimal results. Limited by simple searching behaviors and communication abilities, classical heuristic optimization algorithms are easily stuck to local minima and therefore stop searching for better solutions in the problem space [29]. This phenomenon is referred to as premature convergence, which either leads to poor classification performance or results in the discovery of poor quality feature subsets [30]. Hierarchical heterogeneous particle swarm optimization (HHPSO), as a recently developed variation of PSO, maintains a high level of population diversity during the search and alleviates premature convergence problems by performing diverse searching behaviors [31]. As the success of HHPSO has demonstrated its strength in addressing high-dimensional and complex optimization problems, in this paper, we combine HHPSO with a linear support vector machine (HHPSO-SVM) to perform feature subset selection and classification tasks.

In this paper, extracting discriminating voxel-based brain response patterns that distinguish different cognitive states is a major goal. In MVPA of fMRI data, functional connectivity between individual voxels plays a pivotal role in distinguishing different cognitive states because they capture temporal dependency or causality between different brain regions [15, 17]. However, in existing fMRI analysis, functional connectivity patterns are not intensively analyzed as a whole due to an exponential increase in size of the search space. For this purpose, we develop a new feature interaction detection framework (FIDF) that focuses on identifying informative voxels and voxel-based functional connectivity in two sequential stages. The proposed HHPSO–SVM feature selection approach is implemented in this framework, which is first used to select informative voxels and then used to select a connectivity pattern. The well-known Haxby’s dataset [32] is used to evaluate the effectiveness of the proposed approach.

The rest of this paper is structured as follows. In Sect. 2, the MVPA concept of fMRI data is presented with an explanation of the Haxby’s dataset. In Sect. 3, PSO and HHPSO with their applications are introduced. In Sect. 4, the FIDF using a HHPSO–SVM feature selection algorithm is proposed. Experimental results are presented in Sect. 5. In Sect. 6, this work is concluded with discussions and future work.

## Multi-voxel pattern analysis of fMRI data

Most of previous studies on fMRI data analysis are focused on univariate statistics considering the activity of individual brain locations. Recently, some studies have revealed that the cognitive states of the brain arise in a distributed way over the activity patterns of different regions [32–34]. MVPA can be defined as the general name of the variety of machine learning and pattern recognition techniques to understand neural correlates of cognition by using fMRI data. MVPA has been widely used for decoding the human cognition besides some other applications such as lie detection [35] and memory search [36]. Application of MVPA on resting state fMRI has successfully extracted enough information to detect individual’s brain maturity across development [2]. Also whole-brain resting state functional connectivity patterns of depressed patients are investigated using MVPA to identify the pathological mechanism of major depression [37].

MVPA studies for cognitive state decoding by using fMRI are implemented in three steps: feature extraction, feature selection, and classification [13]. fMRI data for task-based analysis is a plethora of noisy time series measurements. It is usually important to filter out the noise and extract the useful information from this bulky data. In the feature extraction step, voxel responses for each stimulus conditions are mapped onto predefined standard hemodynamic response functions (HRF) and estimated the similarity indexes. This is achieved usually with two ways which are taking the average of the response across time to each stimulus condition and fitting a general linear model (GLM) to a standard hemodynamic response function (HRF) [38]. GLM provides a more representative value about the response of a voxel to the stimulus condition [39].

Feature (voxel) selection plays a vital role in MVPA. In this step, we aim to select a subset of informative voxels features in order to enhance the classification accuracy and/or provide to neuroscientists more refined characteristics of brain functional responses. This task can be done according the predefined region of interest (ROI) identification based on the anatomical structure information in the brain [32]. Or it aims to choose voxels that are significantly active to stimuli by using univariate statistical tools such as ANOVA or *t*-test [40]. In addition, to score voxels according to their individual accuracy level in the experimental settings [40], mutual information [41] and partial least square regression [13] were also used for feature ranking and selection in the literature. Other than these univariate measures, recursive feature elimination is also applied as a multivariate technique to select voxels [10], but the interactions among voxels are not clear yet. Searchlight accuracy based on the neighboring voxels’ contribution to classification for selecting the voxels is also a multivariate technique that considers spatial closeness of the voxels [40]. To the best of our knowledge, the interactions among the voxel activities have not been fully investigated yet in MVPA.

### Haxby’s experiment of visual function

**Fig. 1:**
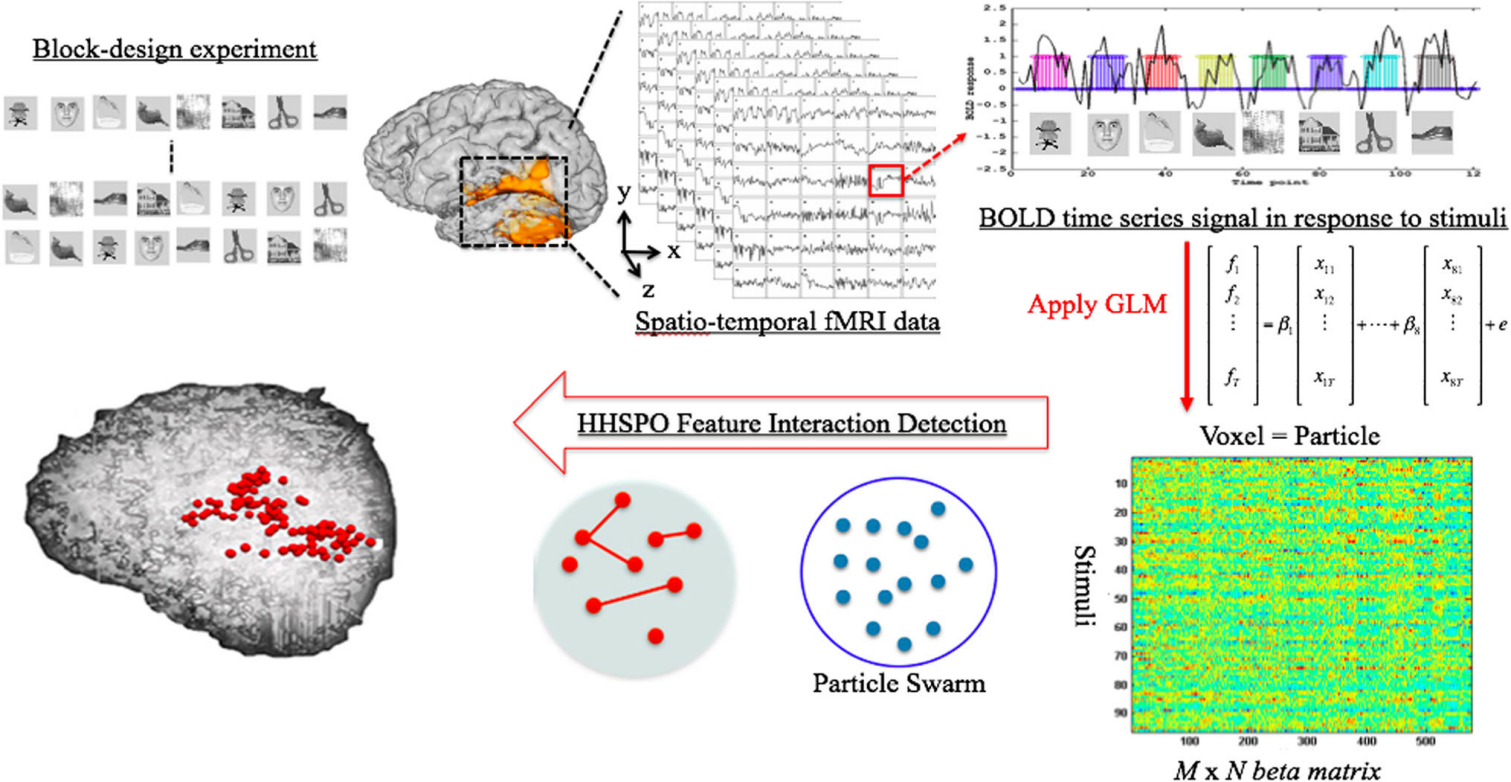
An illustration of the proposed approach to response pattern identification from which a block-design experiment is carried out to examine visual function of fMRI data. Representative features are extracted by applying GLM to BOLD time series across all voxels in ventral temporal cortex in response to eight different stimuli. The feature interaction detection framework is applied to identify discriminating connectivity patterns of selected informative voxels

In this study, we use a benchmark dataset (of six subjects) experimented by Haxby’s research group for experimental tests [32]. In Haxby’s block-design experiment, each subject contains 12 fMRI runs; in each run, eight stimulus blocks, each displaying image exemplars from a different conceptual category were displayed to the subject in a random order, as described in Fig. 1 (upper left). The fMRI data were collected from a GE 3T scanner. One image of brain activity in the dataset (consisting of 64 $\times$ 64 $\times$ 40 voxels) was acquired every repetition time (TR) of 2.5 seconds. Thus, there are a total of 9 TRs (=22.5/2.5) in each block, yielding 720 data instances for the dataset (12 runs $\times$ 8 blocks $\times$ 9 TRs). In our study, we only focused on the predetermined region (region of interest, ROI) of thresholded voxels with task-related variance in the ventral temporal cortex, as opposed to the whole-brain space (around 20,000–40,000 voxels).

To characterize the temporally evolving BOLD signal change in response to a stimulus, a general linear model (GLM) is applied, and coefficient parameters $\beta$ are estimated by fitting a GLM with different predictors for each stimulus block. In this study, the predictors (i.e., $s_{i1}, s_{i2},\ldots , s_{iT}$ for stimulus condition *i* = 1 to 8, and BOLD responses at time 1 to *T*) were modeled with a boxcar convolved with a canonical HRF [42]. We used a double-Gamma function provided by SPM [43], with the default settings, as the HRF. The $\beta$ weights (parameters) are extracted for each run of the experiment, each generating a 3-dimensional $\beta$ weight matrix for each voxel, which can be in turn transformed to a 2-dimensional feature matrix. We denote this input feature matrix *F*, whose size is $M \times N$, where *M* is the number of data instances (the total number of presented stimuli) and *N* is the number of features (voxels). The element $f_{dj}$ of the data matrix *F* represents the real-valued coefficient parameter $\beta$ of the *d*-th data instance at the *j*-th voxel. It is helpful to view $f_{dj}$ as the *d*-th sample of the *j*-th feature random variable $F_{j}$, the *j*-th column of *F*. It is more convenient to treat $F_{j}$ as a random variable of the real-valued coefficient $\beta$ in relevant probabilistic measures. We denote class label $c_{i}\in \{1,\ldots ,K\}$ (i.e., stimulus category), where *K* is the total number of stimulus categories. For each data instance *i*, $c_{i}$ is known precisely according to the experiment design. Figure 1 illustrates the framework to extract features from fMRI signals in the ventral temporal cortex in this case.

## Hierarchical heterogeneous particle swarm optimization

### PSO

Particle swarm optimization (PSO) is a population-based meta-heuristic, originally introduced by Kennedy and Eberhart [44]. Inspired by collective behaviors of bird flocks and fish schools, a PSO algorithm is made of a population of particles. Particles fly through a high-dimensional continuous solution space to find a best solution [45]. During the search, particles iteratively develop their velocities and positions based on their previous best experiences and the global best position in the swarm using Eqs. (1) and (2) as follows:

1 $$V_{i, j}^{t+1} = V_{i, j}^{t} \times \omega +c_{1} r_{1, i, j}^{t}\left( \hat{y}_{j}^{t} -x_{i, j}^{t} \right) +c_{2} r_{2, i, j}^{t}\left( y_{i, j}^{t} -x_{i, j}^{t} \right)$$

and

2 $$x_{i, j}^{t+1}= x_{i, j}^{t} + V_{i, j}^{t+1},$$

where $V_{i, j}^{t}$ denotes the velocity of particle *i* at time *t*, $x_{i, j}^{t}$ is the particle *i*’*s* current position at time *t*, $y_{i, j}^{t}$ is the personal best solution of particle *i* at time t, and $\hat{y}_{j}^{t}$ is the global best solution obtained at time *t*. Subscript *j* is the index of the spatial dimension. $\omega$ is a parameter called inertia weight representing how much the particle’s memory can influence the new position. $c_{1}$ and $c_{2}$ are two constant acceleration coefficients and $r_{1, i, j}^{t}$ and $r_{2, i, j}^{t}$ are two random numbers. They are used to balance exploration and exploitation search behaviors.

### HHPSO

Even though the algorithm design of PSO is simple and computationally efficient, standard PSO is easily trapped into local minima, especially when the optimization problem is complex. In recent years, many variations of PSO have been proposed to overcome this premature convergence problem.

We have recently proposed HHPSO [31]. Compared to standard PSO, the swarm is equipped with multiple equally sized layers. During the search, particles dynamically arrange themselves in a hierarchical structure based on their current fitness values. The better the fitness is, the higher the position in the hierarchical structure is. In HHPSO, particles are not only attracted toward their personal best and global best positions, but they are also attracted toward attractors. For particles in the top layer, their attractors are particles in the same layers with better fitness. For the rest of particles (not in the top layer), their attractors are particles in their immediate superior layer. Herein, a particle’s new velocity is a cumulative effect of (a) its previous velocity, (b) its personal best position, (c) the global best position, and (d) positions of its attractor, as shown in Eq. (3).

3 $$\begin{aligned} \begin{array}{ll} V_{i, j}^{t+1} = V_{i, j}^{t} \times \omega +c_{1} r_{1, i, j}^{t} \left( \hat{y}_{j}^{t} - x_{i, j}^{t} \right) + c_{2} r_{2, i, j}^{t} \left( y_{i, j}^{t} -x_{i, j}^{t} \right) \\ \qquad\quad+\sum\limits _{a = 1}^{A_{i}^{t}} c_{3} r_{3, i, j}^{t}\left( x(i)_{a, j}^{t} -x_{i, j}^{t} \right) , \end{array} \end{aligned}$$

where $x(i)_{a,j}^{t}$ is the position of attractor particle *a* of particle *i* in dimension *j* at time *t*. $A_{i}^{t}$ is the total number of attractors of particle *i* at time *t*. $c_{3}$ is a constant acceleration coefficient and $r_{3, i, j}^{t}$ is a random number. Other parameters are exactly the same as those used in Eq. (1).

For the searching behavior, in HHPSO algorithm, particles are allowed to perform different searching behaviors based on their ranks in the hierarchy and their current performances. For example, if a signal of premature convergence (i.e., early stagnation or overcrowding) is detected, the relevant particle will change its previously adopted searching behavior and randomly select a new searching behavior from the predefined behavior pool to avoid premature convergence [31]. Compared to standard PSO, HHPSO is more resistant to local minima and superior to sustain the population diversity as the dimension of the search space grows.

Recently, PSO as well as its variations has been implemented as efficient global optimization techniques, which received considerable attention in machine learning (ML), data mining, and pattern recognition [46–48]. These algorithms have shown to perform very well on algorithm development and parameter optimization tasks [49–52].

## HHPSO–SVM for voxel selection in MVPA

### Problem definition

HHPSO–SVM feature selection algorithm (HHPSO–SVM) aims to maximize classification accuracy (Max-Accuracy) and to minimize the size of selected features (Min-Size) simultaneously. The objective function in Eq. (5), which is utilized to quantify searched solutions, is defined by dividing the classification error by the number of eliminated features. The penalization term (i.e., Min-Size) is used for the purpose of constructing a compact set of features and controlling overfitting. The approach iterates until a best solution (a subset of features) is found.

4 $$f = \max {\big \langle Accuracy(S_{i})\big \rangle } \min {\big \langle Size (S_{i})\big \rangle }$$

5 $$\begin{aligned} {f_{i}} = \frac{{Error(S_{i})}}{{N- Size(S_{i})}} \end{aligned}$$

In Eqs. (4) and (5), $S_{i}$ represents the feature subset selected by particle *i*. $Accuracy(S_{i})$ and $Error(S_{i})$ represent the classification accuracy and error calculated by using feature subset *i*. *N* is the entire number of features. $Size(S_{i})$ represents the number of features in subset *i*.

### Algorithm design

In the HHPSO–SVM feature selection algorithm, HHPSO provides multiple candidate solutions to feature selection and SVM is employed to evaluate the classification performance using these candidate solutions. Particles cooperate to locate a best solution in an *N* dimensional problem space, where *N* is the cardinality of the original feature set. Positions of particles are represented as numeric strings of length *N*. Each value in the string is within zero and one, which can be seen as the contribution of the corresponding feature to the classification task. The higher the value, the more important it is. Each particle selects a set of important features based on its position string.

Each iteration involves two steps (see Algorithm 1). In the first step, we identify the selected features and evaluate the fitness value for each particle (lines 1–10). Taking particle *i* for an example, a predefined threshold $\theta$ is applied to its current position $x_{i}$. The *j*-th feature will be selected, if the *j*-th value in the position string is greater than $\theta$. With the selected features, classification error as well as the number of eliminated features is calculated by SVM to evaluate the fitness value for particle *i* (see Eq. 5). After all particles finish updating their fitness values and their personal best solutions, the global best solution is defined by the best of the personal best solutions in the swarm.

In the second step, particles are ranked by their fitness values in an ascending order and directed to the right layer in the hierarchical structure (lines 11–15). Based on the rank, particles occupy layers from top to bottom. Particles in the higher layers always have better fitness values than particles in the lower layers.

In the third step, particles update their velocities and positions based on their searching performances as well as their positions in the hierarchical structure (lines 16–25). This step ensures that the swarm continuously explores the problem space and optimizes solutions iteration by iteration.

The algorithm terminates when it converges to a stationary solution, which is defined by a condition that the global best position stops to evolve for more than 50 iterations. As the algorithm converges, the final solution to feature selection is obtained by applying the threshold ($\theta$) to the global best position (line 26).

In HHPSO–SVM (Algorithm 1), *P* represents the swarm population, and $P_{i}$ represents particle *i*. *n* is the number of particles in the swarm. *N* is the dimension of the problem space. $x_{i, j}$ is particle *i*’s current position in dimension *j*. $S_{i}$ denotes the subset of features selected by $P_{i}$. $F_{j}$ represents the *j*-th feature in the original feature space. $y_{i}$ represents the personal best position of particle *i* at time *t*. $\hat{y}$ represents the global best position. *f* represents the fitness function. $R_{i}$ represents the *i*-th particle in the swarm, after sorting all particles by their fitness values in an ascending order. $L_{o}$ represents the first layer and $L_{j}$ represents the $(j+1)$-th layer. *l* and *k* are the number of layers and the number of particles in a layer, respectively. $A_{i}$ denotes the set of attractors of $P_{i}$.

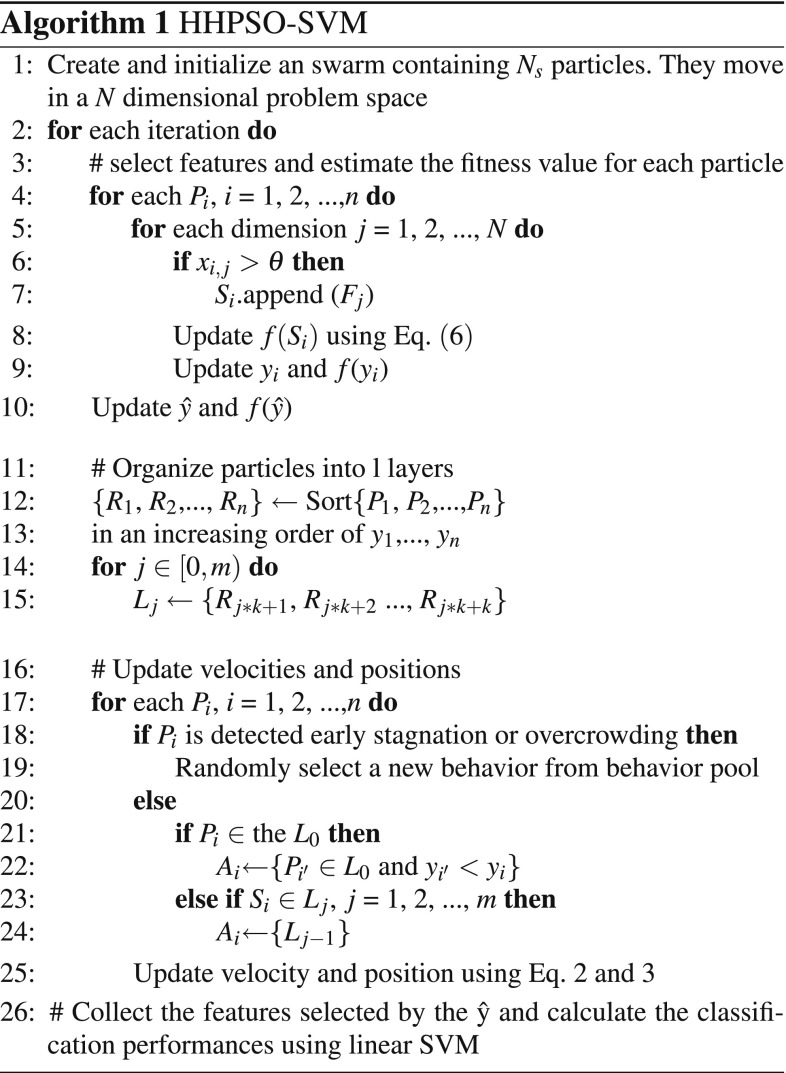



### Feature interaction detection framework

In order to extract discriminating multi-voxel patterns from fMRI data, scalable, robust, and efficient dimension reduction tools are desired to identify influential voxels and voxel-based connectivity. In this paper, FIDF is developed as a MVPA approach that undergoes a two-stage procedure. Voxel selection (feature selection) and voxel connectivity selection (feature interaction selection) are performed in Stage I and Stage II, separately. The proposed HHPSO–SVM is adopted as the feature selection method under this framework.

In the first stage, the feature selection algorithm is implemented to select the best subset of voxels. This procedure is repeated 15 times to obtain the average number of selected voxels ($N_{avg}$) and frequencies of voxels being selected. Voxels are ranked according to their selection frequencies in a descending order. The top $N_{1}$ ($N_{1}\, =\, 1.05 N_{avg}$) voxels are selected in Stage I. In the second stage, we first establish all connectivity that connects voxels selected in Stage I, which is equivalent to constructing a fully connected network. In this stage, we aim at extracting discriminating connectivity patterns from a fully connected structure. The rationale is as follows: HHPSO–SVM selects a best combination of voxels in the first stage, which means the selected voxels are interactive and informative as a combination. Identifying consistent connectivity patterns from the pre-selected voxel combination may achieve similar or even better classification performances than only considering individual voxels.

For fMRI data, the connectivity between two voxels is generated via finding products of all pairs of voxels. This type of connectivity definition is similar to using correlation coefficients, mutual information, or consistency measures to quantify the connectivity between two voxels. By doing this, the dimension of feature space becomes $N_{1} (N_{1}-1)/2$. HHPSO–SVM is implemented again to select the best subset of connectivity that distinguishes multiple classes. Similarly, this algorithm is repeated 15 times to identify robust connectivity patterns.

In the present study, we utilize a 12-fold cross-validation to assess the performance of different feature selection algorithms. We first divide the whole data into 12 portions of equal size. The optimization procedure is performed on the 11 portions of data, and the remaining 1 fold is held out to evaluate the algorithm’s performance. During optimization, the training set is further randomly split, in which 6 portions are used to train the model and the other 5 portions are used to test the results. The random splitting is repeated 20 times, and the average classification error rate and the average number of selected feature subsets are used to estimate the fitness function.

The final decision of feature selection is determined by the global best solution obtained at the end of optimization. The same threshold ($\theta$) and mechanism are applied to select a robust set of connectivity features. The classification performance is examined on the holdout dataset. Means and standard deviations are computed using this 12-fold cross-validation approach.

## Experimental analysis

### Experimental setting

Comparative experiments were carried out for the Haxby’s dataset [32]. For a comparison purpose, the same data preprocessing techniques, including using z-score to standardize the data and randomly shuffling the original data matrix, were applied to attenuate noise and improve spatial alignment of time series data [53].

The performance of the proposed HHPSO–SVM selection algorithm was evaluated by comparing it with (a) without feature selection (WFS), (b) sequential forward feature selection (SFS), (c) sequential backward feature selection (SBS), and (d) standard PSO feature selection algorithm (PSO–SVM). SFS and SBS are deterministic greedy algorithms and can only produce a single solution for each dataset. PSO–SVM combines standard PSO and linear SVM, and it adopts the same objective function to explore the best solution to feature selection. The mechanism of PSO–SVM is similar to HHPSO–SVM. All five algorithms were applied to FIDF to select voxels in the first stage and select voxel-based connectivity in the second stage.

In this study, both HHPSO–SVM and PSO–SVM employed a swarm containing 50 particles. The acceleration coefficients, $c_{1}$ and $c_{2}$, are linearly changed over time. $c_{1}$ linearly decreased from 2.5 to 0.5, and $c_{2}$ linearly increased from 0.5 to 2.5 using the formula shown in Eqs. (6) and (7), where $n_{t}$ is the overall iteration time, and *t* is the current iteration, as follows:

6 $${c_{1}(t)}= {(c_{1, \mathrm {min}}} - {c_{1, \mathrm {max}})} \frac{t}{n_{t}} + c_{1, \mathrm {max}}$$

and

7 $${c_{2}(t)}= {(c_{2, \mathrm {max}}} - {c_{2, \mathrm {min}})} \frac{t}{n_{t}} + c_{2, \mathrm {min}}.$$

**Fig. 2:**
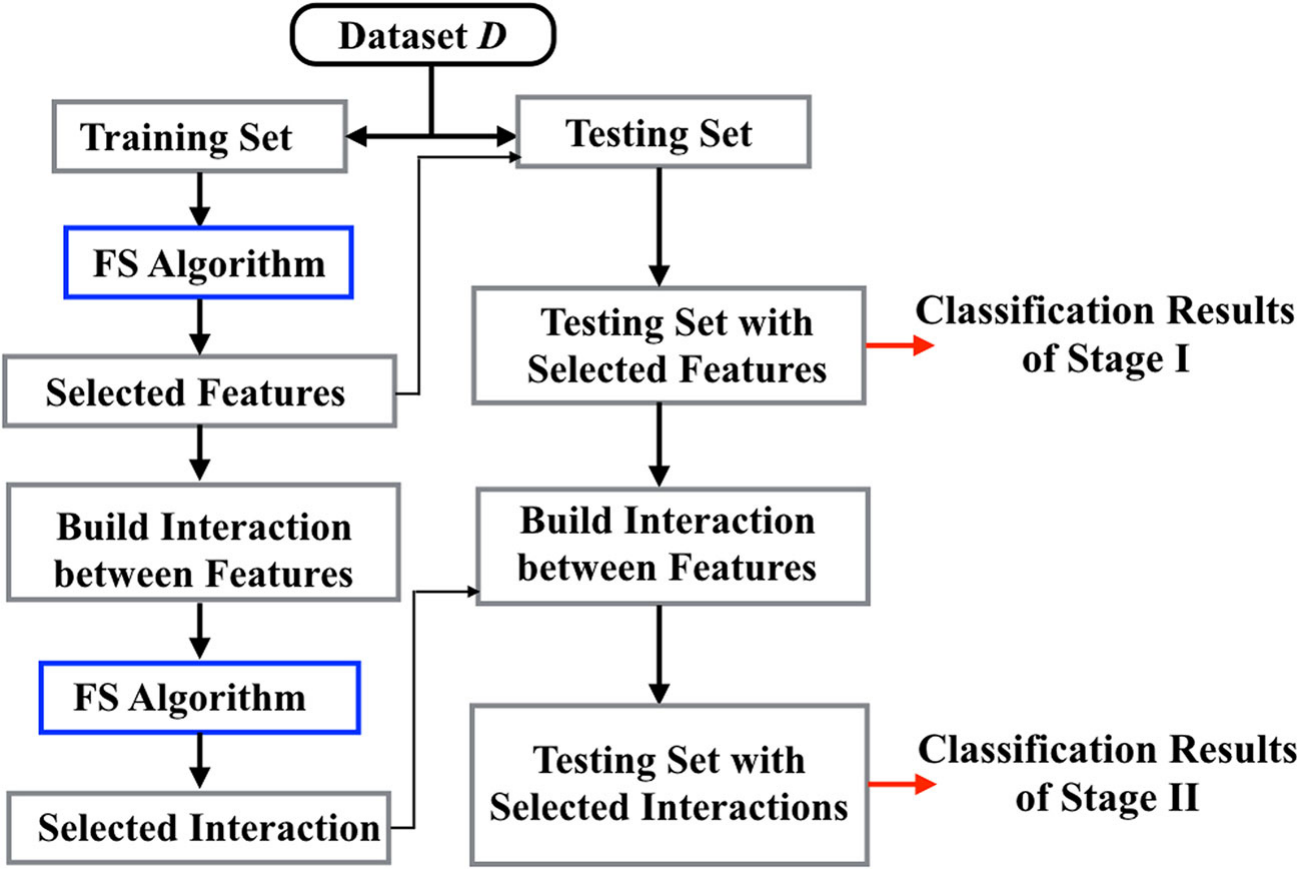
A conceptual flowchart of the proposed feature interaction detection framework. FS Algorithm stands for feature selection algorithm

We implemented the linear support vector machine (SVM) from the scikit-learn in Python, with the parameter *c* set to 1 in all experiments [54]. For both PSO and HHPSO, the value of threshold ($\theta$) is 0.95. For HHPSO, the hierarchical population structure consisted of five layers as shown in Fig. 2.

### Experimental results

**Fig. 3:**
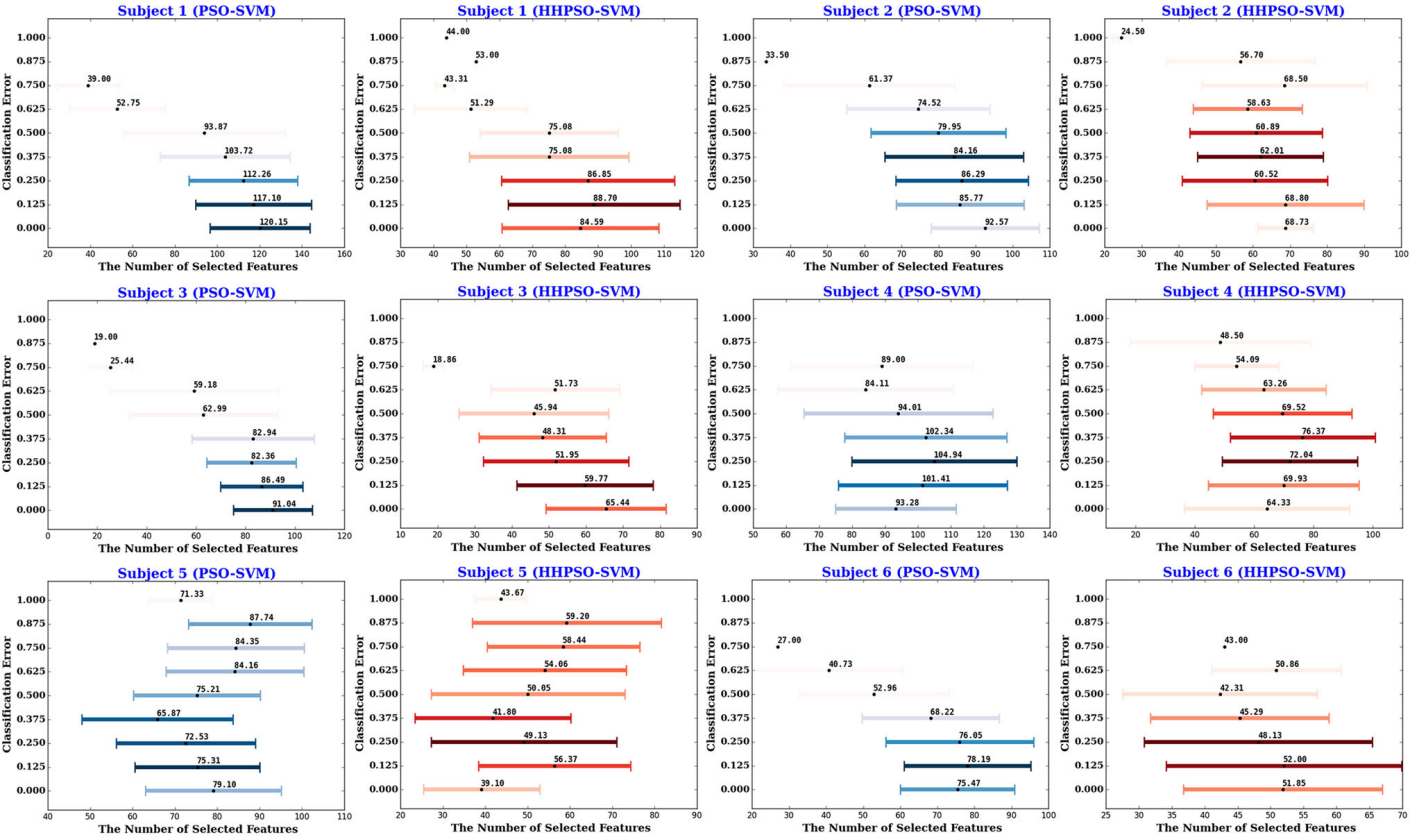
Cross-validated solutions of PSO–SVM (*in blue*) and HHPSO–SVM (*in red*) from Stage I, where *x*-axis represents the number of selected voxels and *y*-axis represents the classification error. Lighter color means that the solution is obtained in earlier optimization iterations, while darker color denotes the solution is obtained in later optimization iterations. (Color figure online)

**Fig. 4:**
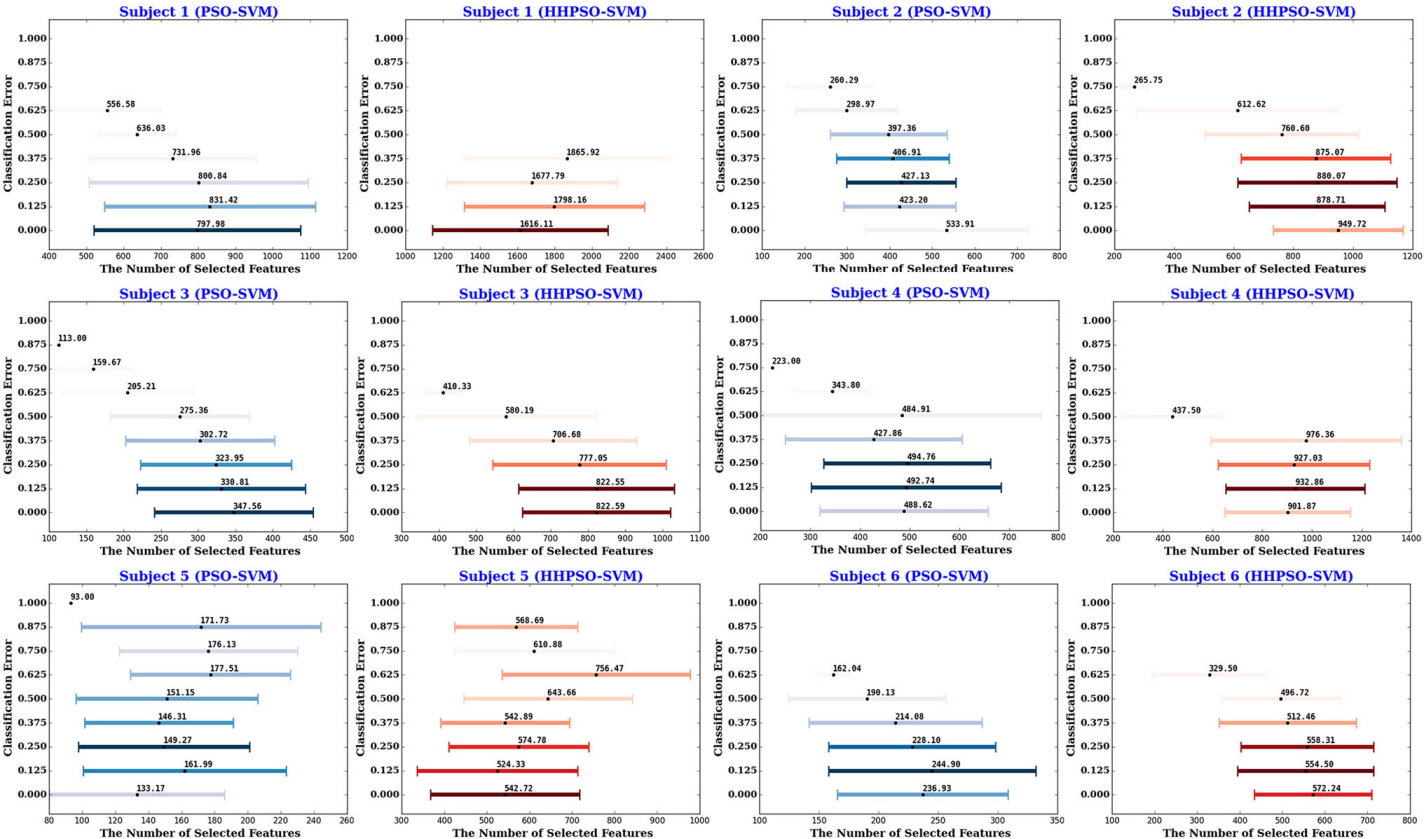
Cross-validated solutions of PSO–SVM (*in blue*) and HHPSO–SVM (*in red*) from Stage II, where *x*-axis represents the number of selected voxels and *y*-axis represents the classification error. Lighter colormeans that the solution is obtained in earlier optimization iterations, while darker colordenotes the solution is obtained in later optimization iterations. (Color figure online)

The comparative classification results of the five different algorithms are summarized in Tables 1 (Stage I) and 3 (Stage II). The statistics of the number of selected features are presented in Tables 2 and 4 for Stage I and Stage II, respectively. For PSO–SVM and HHPSO–SVM, the distributions of their obtained solutions from Stage I and Stage II are visualized in Figs. 3 and 4. Finally, we compared our results using FIDF and HHPSO–SVM with the results published in [53], the comparison results are shown in Table 5.

In Stage I, HHPSO–SVM feature selection algorithm exhibited the highest classification accuracy for subjects 1, 3, and 4. WFS achieved the best classification accuracy for subjects 2, 5, and 6. Compared to the results obtained by WFS, HHPSO–SVM and PSO–SVM yielded comparably good classification results for subjects 2 and 6. However, for subject 5, the classification results produced by HHPSO–SVM and PSO–SVM were not as good as the results produced by WFS or SBS.

**Table 1:** Classification results of Stage I of FIDF

Stage I	WFS		SFS		SBS		PSO–SVM		HHPSO–SVM	
	Mean	SD	Mean	SD	Mean	SD	Mean	SD	Mean	SD
Sbj 1	0.875	0.191	0.693	0.153	0.875	0.190	0.819	0.130	0.885	0.099
Sbj 2	0.708	0.106	0.517	0.140	0.708	0.106	0.623	0.141	0.696	0.143
Sbj 3	0.864	0.148	0.686	0.161	0.865	0.148	0.792	0.140	0.874	0.118
Sbj 4	0.677	0.148	0.560	0.150	0.677	0.148	0.676	0.155	0.708	0.186
Sbj 5	0.705	0.312	0.568	0.202	0.685	0.323	0.562	0.270	0.614	0.270
Sbj 6	0.875	0.125	0.684	0.180	0.875	0.125	0.805	0.122	0.852	0.104

The classification accuracy and standard deviations of *WFS* without feature selection, *SFS* sequential forward feature selection, *SBS* sequential backward feature selection, *PSO–SVM* and *HHPSO–SVM* were calculated for subject 1 to subject 6

The average number of features selected by each algorithm has been presented in Table 2. HHPSO–SVM selected 20–30 % of features, while PSO–SVM selected 10–20 % percent of features in Stage I. Both of them reduced the dimension of feature space considerably. However, SFS and SBF failed to add/eliminate features after few iterations, which means that SFS only included few features, and SBF almost included all features as shown in Tables 3, 4 and 5.

**Table 2:** The number of selected voxels in Stage I of FIDF

Stage I	SFS		SBS		PSO–SVM		HHPSO–SVM	
	Mean	SD	Mean	SD	Mean	SD	Mean	SD
1	39	5	577	0	84	24	116	22
2	30	5	464	0	60	15	84	15
3	32	5	306	1	55	17	88	16
4	31	5	675	0	68	21	100	20
5	30	5	420	0	38	13	54	13
6	34	4	348	1	55	16	82	14

Average number and standard deviation of selected voxels are calculated for *WFS* without feature selection, *SFS* sequential forward feature selection, *SBS* sequential backward feature selection, *PSO–SVM* and *HHPSO–SVM*

**Table 3:** Classification results of Stage II of FIDF

Stage II	WFS		SFS		SBS		PSO–SVM		HHPSO–SVM	
	Mean	SD	Mean	SD	Mean	SD	Mean	SD	Mean	SD
1	0.792	0.191	0.389	0.149	0.802	0.194	0.922	0.107	0.948	0.081
2	0.625	0.135	0.274	0.125	0.615	0.126	0.694	0.132	0.796	0.126
3	0.813	0.207	0.333	0.171	0.823	0.119	0.846	0.143	0.874	0.119
4	0.604	0.100	0.363	0.134	0.552	0.101	0.803	0.116	0.847	0.101
5	0.647	0.270	0.297	0.170	0.545	0.284	0.600	0.253	0.714	0.284
6	0.760	0.139	0.451	0.171	0.730	0.122	0.819	0.133	0.840	0.122

The classification accuracy and standard deviations of *WFS* without feature selection, *SFS* sequential forward feature selection, *SBS* sequential backward feature selection, *PSO–SVM* and *HHPSO–SVM* were calculated for subject 1 to subject 6

**Table 4:** The number of selected features in Stage II of FIDF

Stage II	SFS		SBS		PSO–SVM		HHPSO–SVM	
	Mean	SD	Mean	SD	Mean	SD	Mean	SD
1	26	5	166176	0	790	251	1704	438
2	16	4	107416	0	402	115	887	218
3	20	3	46665	0	329	95	815	196
4	26	4	227475	0	467	158	884	250
5	43	8	87990	0	153	53	560	164
6	29	4	60378	0	232	69	546	139

Average numbers and standard deviations of selected features are calculated for *WFS* without feature selection, *SFS* sequential forward feature selection, *SBS* sequential backward feature selection, *PSO–SVM* and *HHPSO–SVM* are presented

**Table 5:** A classification performance comparison between FIDF and a feature selection framework using mutual information (MI) and partial least square regression (PLS) published in [27]

Study	Subject 1	Subject 2	Subject 3	Subject 4	Subject 5	Subject 6
Previous study	0.940	0.780	0.860	0.800	0.720	0.880
Our study	0.948	0.796	0.874	0.847	0.714	0.840

In Stage I, HHPSO–SVM and PSO–SVM successfully reduced the number of selected features, therefore the computational complexity of Stage II was significantly reduced. Implementing WFS and SBF in Stage II was computationally expensive. Compared to results of Stage I, PSO–SVM and HHPSO–SVM improved their classification accuracy remarkably in Stage II. For subjects 1, 2, 4, and 5, the average classification accuracy increased around 10 %. However, SFS and SBS performed the classification task with significant degradation in accuracy. One possible reason is that greedy iterative optimization algorithms consider features one-by-one for addition/removal, so that the algorithms may easily get stuck into local minima when the dimension of data is high.

In Stage II, HHPSO–SVM outperformed all other algorithms for all subjects in terms of classification accuracy. Regarding the number of selected connectivity, HHPSO–SVM selected less than 20 % of connectivity. Even though PSO–SVM selected less connectivity than that of HHPSO, the algorithm yielded significantly lower classification accuracy. Both SFS and SBS failed to find discriminating connectivity among their pre-selected informative voxels.

We visualized historical solutions obtained by PSO–SVM and HHPSO–SVM in Stage I (Fig. 3) and Stage II (Fig. 4) over time. The results provided an estimate of how well the two algorithms balance the trade-offs between accuracy and feature simplicity during the optimization process. In these figures, color is used to represent how many iterations an algorithm takes to obtain that solution. Darker color means longer iterations. The distribution of historical solution illustrates HHPSO–SVM offered significantly better trade-offs between accuracy and feature simplicity. Compared to PSO–SVM, HHPSO–SVM obtained higher classification accuracy using a smaller subset of features.

Finally, we compared our final classification results to results published in [53], which combines mutual information (MI) and partial least square regression (PLS) to select features. The comparison results showed that our approach produced better classification results for subjects 1, 2, 3, and 4. However, for subjects 5 and 6, our results were slightly worse than their best results.

## Conclusions and discussion

In this paper, we addressed and solved the challenging, high-dimensional voxel selection problem in MVPA in neuroscience by combining HHPSO and SVM. Compared to the classification results obtained by four other algorithms, including WFS, SFF, SBF, and PSO–SVM, our proposed HHPSO–SMV led to two advantages: (1) it quickly removed the irrelevant and redundant features, and (2) HHPSO–SVM feature selection algorithm outperformed other algorithms in terms of classification accuracy. Compared to PSO–SVM, feature selection results obtained by HHPSO–SVM achieved better trade-offs between accuracy and feature simplicity, which indicated the importance of maintaining a high level of population diversity and performing appropriate searching behaviors to heuristic optimization. Processing these properties, HHPSO–SVM feature selection algorithm is robust in tackling high-dimensional feature selection tasks.

The proposed FIDF successfully extracted discriminating voxel-based connectivity patterns from high-dimensional fMRI datasets. This framework, which focused on finding a subset of interacted features (or voxels) in the first stage and further eliminated interaction (or connectivity) redundancy in the second stage, yielded improved classification results. Identifying the functional connectivity patterns from a set of pre-selected voxels provided valuable insights for brain response pattern identification. Implementing this framework, the classification performances were further improved for most subjects. Its simplicity and ease of implementation have been demonstrated.

However, the proposed approach is still  faceed with some challenging issues. For example, the proposed HHPSO–SVM feature selection algorithm requires properly tuning parameters, e.g., the number of layers and the value of threshold $\theta$. A hierarchical structure with five layers is designed for a swarm that contains fifty particles, and the selected threshold ($\theta$ = 0.95) is determined based on the previous experiments. There is no proof that the selected values are the best choices. A systematic study regarding the sensitivity and effectiveness of different parameter settings needs to be undertaken. Future work will emphasize on analysis and interpretation of identified brain response patterns. In addition, a thorough comparison of the proposed algorithm with other brain response pattern identification tools will be conducted.
